# Supplementary material for: Sorption–Biological Treatment of Coastal Substrates of the Barents Sea in Low Temperature Using the Rhodococcus erythropolis Strain HO-KS22
Source: Microorganisms. 2025 Sep 18;13(9):2181. doi: 10.3390/microorganisms13092181 (PMC12472628; doi:10.3390/microorganisms13092181)
Supplement: Supplementary file 1 [file microorganisms-13-02181-s001.zip › microorganisms-3818668-supplementary.pdf]

Supplementary Materials:

Table S1. Total petroleum hydrocarbon content (mean±SE), mg·kg^{-1}

	Light oil				Medium oil				Heavy oil			
	**1**	**2**	**3**	**4**	**1**	**2**	**3**	**4**	**1**	**2**	**3**	**4**
**Sand**												
Initial	3977±317				1894±137				2248±15			
1 month	3510±247	2518±143	2088±155	2592±343	1794±226	1509±151	1786±210	1329±86	2080±202	1600±47	2059±209	2032±44
2 months	2783±119	2144±321	1997±244	1897±51	1550±98	1050±91	1102±118	931±14	1507±63	1222±139	990±113	1011±112
3 months	1489±130	995±23	1062±25	1018±151	877±139	568±32	578±35	489±40	818±70	646±39	533±72	567±53
**Soil**												
Initial	6979±422				7903±1111				6609±785			
1 month	4684±402	4561±689	5311±663	3946±420	2868±143	3400±307	3372±358	3240±125	3735±3162	3518±405	4073±670	3572±110
2 months	3926±257	3030±465	3813±51	3537±297	2907±265	2490±203	2580±428	2940±100	3544±544	2936±427	3521±183	2606±217
3 months	3407±344	3298±447	3110±369	3448±36	2251±180	2386±201	2128±48	2299±393	2365±67	2586±80	2473±279	2662±2406

Note: 1 – natural attenuation, 2 – *R. erythropolis* and peat, 3 – *R. erythropolis* and activated carbon, 4 – *R. erythropolis* and vermiculite.

Table S2. Content of high-molecular organic compounds (mean±SE), mg·kg^{-1}

	Light oil				Medium oil				Heavy oil			
	**1**	**2**	**3**	**4**	**1**	**2**	**3**	**4**	**1**	**2**	**3**	**4**
**Sand**												
Initial	1372±218				754±109				1218±147			
1 month	1193±87	724±84	652±13	766±55	714±10	637±94	685±65	576±65	1046±38	962±86	1226±158	1016±24
2 months	1169±128	941±114	914±144	834±92	950±97	885±40	856±124	663±15	1411±174	1153±136	1113±167	1083±119
3 months	3146±186	2158±214	1732±199	2214±149	878±33	979±103	525±46	798±107	1683±156	1591±187	1126±85	1000±27
**Soil**												
Initial	2549±117				4659±295				3527±263			
1 month	2452±170	2381±208	2944±213	2181±248	2051±261	2274±350	2164±35	2217±109	3059±395	2818±538	2718±368	2909±285
2 months	2200±153	1730±193	2137±310	1982±234	2075±207	2149±284	1926±193	2499±224	2770±326	2454±84	2925±60	1811±162
3 months	1821±122	1732±88	1530±137	1667±75	1857±198	1659±254	1468±119	1539±163	2421±341	2415±97	2288±106	2559±316

Note: 1 – natural attenuation, 2 – *R. erythropolis* and peat, 3 – *R. erythropolis* and activated carbon, 4 – *R. erythropolis* and vermiculite.

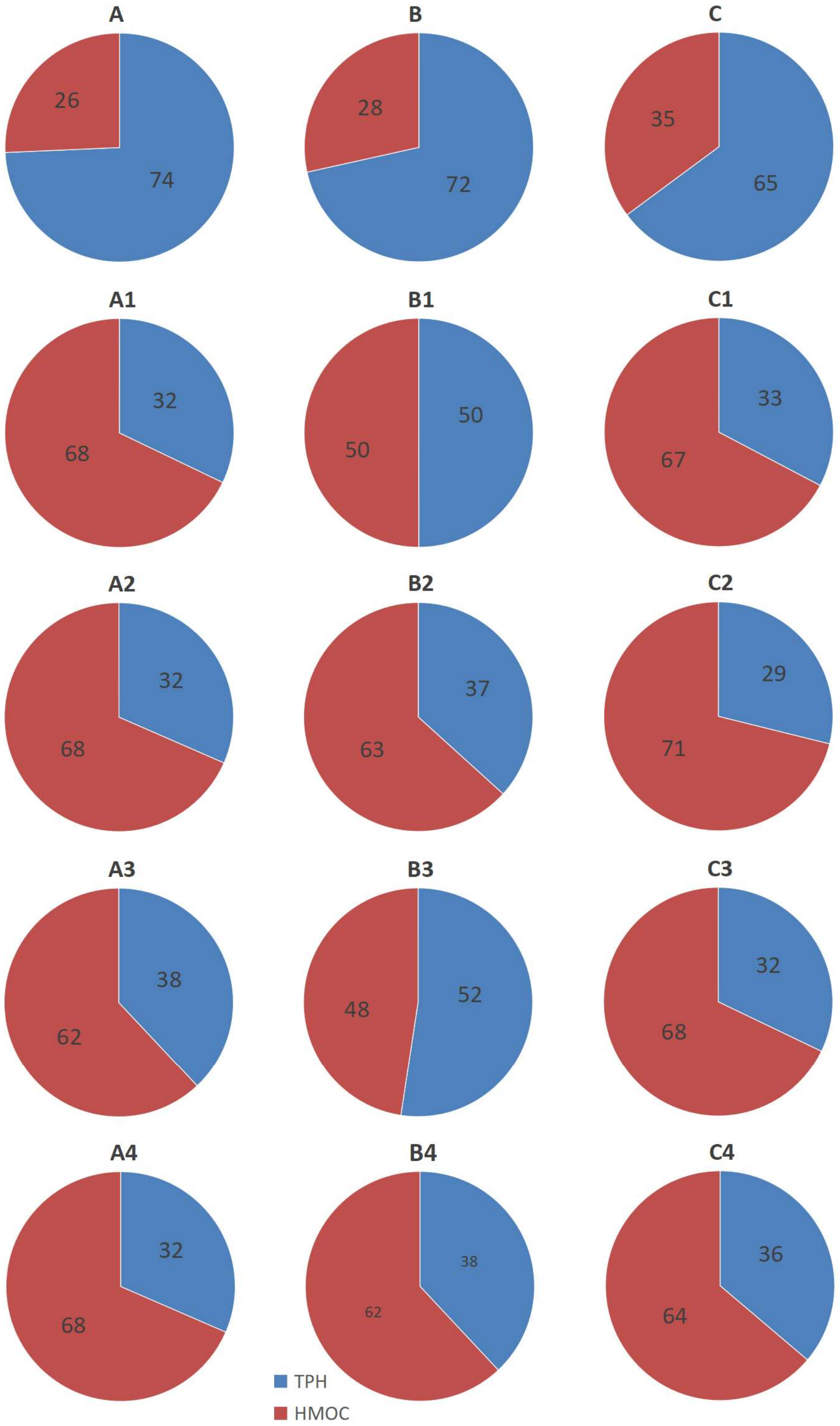

Figure S1. The ratio of TPH to HMOC in the sand with emulsion of light (A), medium (B) and heavy (C) oil before treatment (A-C); after three months of natural attenuation (A1-C1), *R. erythropolis* and peat (A2-C2), *R. erythropolis* and activated carbon (A3-C3), *R. erythropolis* and vermiculite (A4-C4).

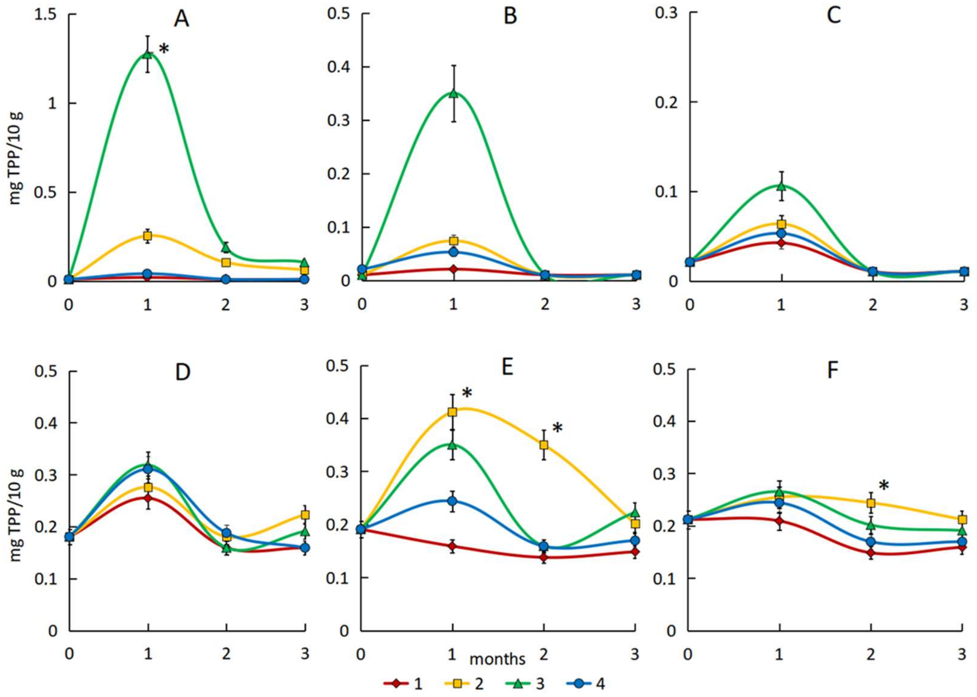

Figure S2. Dehydrogenase activity in the sand with emulsion of light (A), medium (B), heavy (C) oil and in the soil with emulsion of light (D), medium (E), heavy (F) oil. 1 – natural attenuation, 2 – *R. erythropolis* and peat, 3 – *R. erythropolis* and activated carbon, 4 – *R. erythropolis* and vermiculite; * - reliable differences between the natural attenuation and the sorption-biological treatment with a significance level of 0.05.

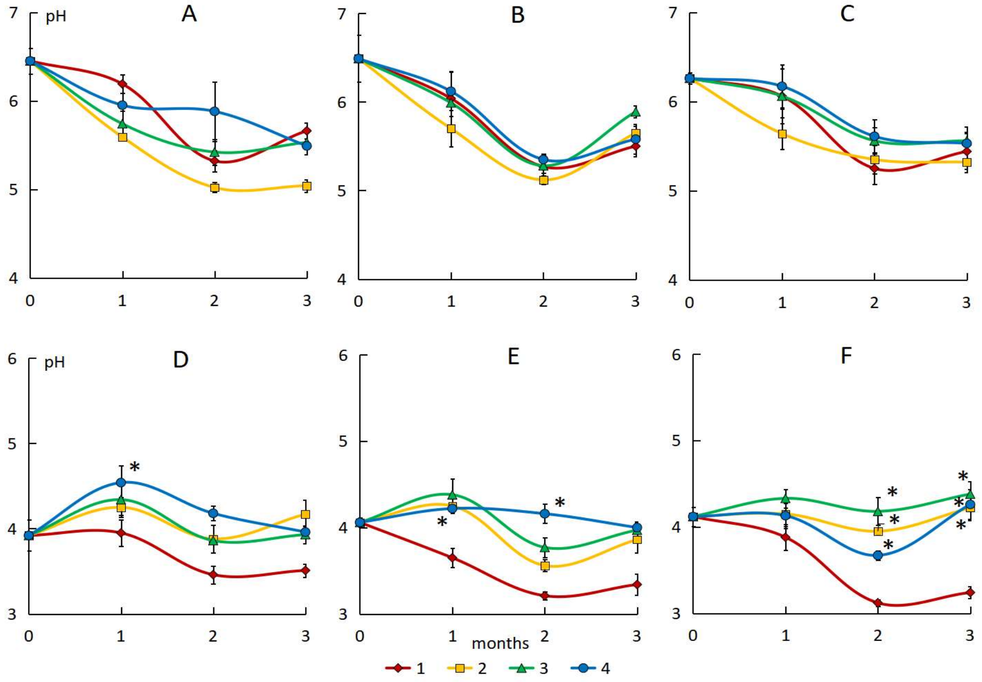

Figure S3. Dynamics of pH value in the sand with emulsion of light (A), medium (B), heavy (C) oil and in the soil with emulsion of light (D), medium (E), heavy (F) oil. 1 – natural attenuation, 2 – *R. erythropolis* and peat, 3 – *R. erythropolis* and activated carbon, 4 – *R. erythropolis* and vermiculite, 5 – background value; * - reliable differences between the natural attenuation and the sorption-biological treatment with a significance level of 0.05.
